# Editorial: Emerging trends and advances in the socioeconomic applications of beneficial microbes

**DOI:** 10.3389/fmicb.2024.1523569

**Published:** 2024-12-10

**Authors:** Laurent Dufossé, Pragya Tiwari

**Affiliations:** ^1^CHEMBIOPRO Laboratory, Department of Food Science & Biotechnology, University of Réunion Island, Saint-Denis, France; ^2^Department of Horticulture and Life Science, Yeungnam University, Gyeongsan, Gyeongbuk, Republic of Korea

**Keywords:** beneficial microbes, socioeconomic applications, emerging trends, advances, benefits for humans, benefits for animals, benefits for plants

Plants interact with a wide range of microorganisms (bacteria, yeasts, filamentous fungi, viruses). Although plant pathogens have received the most attention because of their harmful effects on plant growth, development, and productivity, they represent only a small part of the microbial communities. In contrast, non-pathogenic microorganisms are abundant in Nature and can establish mutualistic relationships with their plant hosts. The United Nations General Assembly (UNGA) Science Summit contemplated, “understanding the world of microbes is imperative either to curb dangerous effects or to harness their power for healthier life, for sustainable energy sources, for biodiversity, for tackling climate change and for solving hunger problems”, one of the key objectives of the United Nations Sustainable Development Goals (SDGs). The initiatives by UN SDGs cointegrate microbial sciences and biotechnologies for a better life and facilitate innovations, health and wellbeing, hunger elimination, clean water and sanitation, industry, and infrastructure, providing sustainable solutions. This editorial discusses the latest advances in microbial biotechnologies and state-of-the-art concepts in harnessing microbial traits for sustainable livelihood, and addressing societal concerns, defining the future trajectory of this emerging field.

The field of plant-microbe biotechnologies is rapidly evolving, and significantly contributing to agriculture, healthcare, and environmental subsistence including other biotechnological innovations. The diverse microorganisms produce different functional foods and other high-value food ingredients, including pigments, enzymes, and food flavors, and are also vital in improving crop yield and productivity in agriculture. In response to global food demand, the beneficial implications of microorganisms in agriculture need to be promoted via the use of microbial inoculants as biofertilizers (and reduce chemical fertilizer usage), soil-carbon restoration, and genetic manipulation studies to harness their full potential. In healthcare, many drugs, including anticancer drugs and antibiotics, are microbe-derived, and microbial platforms are utilized as biofactories for the production of novel drugs and proteins via recombinant DNA technologies. Recent research has also highlighted the importance of gut microorganisms in digesting food components and vitamin production for human health. In the field of environmental management, microorganisms-assisted remediation of contaminated water has been successful in improving water quality and sanitation. Furthermore, microorganisms assist in biofuel production and act as a direct source of clean and affordable energy, produce substances and metabolites of high industrial importance, bioremediate environmental hazards and plastics, and enhance plant productivity and stress tolerance.

Decades of agricultural intensification have boosted crop yields at the expense of soil health and microbial diversity, jeopardizing global food security. To address this Research Topic, a study in West Bengal, India (Mukhopadhyay et al.), explored the potential of a novel multi-strain consortium of plant growth-promoting (PGP) *Bacillus* spp. for soil bioaugmentation. In this work, a composite inoculum of *Bacillus zhangzhouensis* MMAM, *Bacillus cereus* MMAM3, and *Bacillus subtilis* MMAM2 was introduced into an over-exploited agricultural soil and implications on the improvement of vegetative growth and yield-related traits of *Glycine max* (L) Meril. plants were evaluated, growing them as model plant, in pot trial condition. The study's findings demonstrated significant improvements in plant growth and soil microbial diversity when using the bacterial consortium in conjunction with vermicompost. Metagenomic analyses revealed increased abundance of many functional genera and metabolic pathways in consortium-inoculated soil, indicating enhanced soil biological health.

In a second study Debnath et al. aimed to understand plant-bacteria interactions that enhance plant resistance to environmental stressors, with a focus on maize (*Zea mays* L.) and its vulnerability to various pathogenic organisms. The potential of 1-amino-cyclopropane-1-carboxylic acid (ACCA) as a compound to boost maize's resilience against stressors and pathogens is hypothesized through an empirical computational study and needs to be confirmed by biological studies conducted for example in greenhouses.

New microbial strains interacting with plants are isolated every day. The third article (Patakova et al.) of this Research Topic presents new information about the genome and phenotypic characteristics of *Pantoea agglomerans* strain DBM 3797, isolated from fresh Czech hop (*Humulus lupulus*). *P. agglomerans* DBM 3797 was cultured under aerobic and anaerobic conditions, its metabolites were analyzed by HPLC and it was tested for plant growth promotion abilities, such as phosphate solubilization, siderophore, and indol-3-acetic acid productions. In addition, genomic DNA was extracted, sequenced, and *de novo* assembly was performed. Further, genome annotation, pan-genome analysis, and selected genome analyses, such as CRISPR arrays detection, antibiotic resistance, and secondary metabolite genes identification were carried out. As concluded by authors, this strain has a number of properties potentially beneficial to the hop plant, however, its safety profile needs to be addressed in follow-up research.

In another article by Dhar et al., focus is put on *Rhododendron ferrugineum* L., Nepal's national flower and Uttarakhand's state tree, thriving in high-altitude mountain ecosystems. Leaf anomalies were traced back to the pathogenic fungus *Curvularia tuberculata*, marking the first documented case of its impact on *R. ferrugineum* in India. Overall, this study calls for proactive measures to protect *R. ferrugineum's* cultural and ecological heritage and emphasizes the significance of interdisciplinary approaches (including researches to identify a biological control agent able to manage the pathogenic fungus *Curvularia tuberculata*) in addressing emerging ecological threats.

A second computational study is presented in Perveen et al. with investigations on the synergistic action of plant natural products curcumin and mangiferin against *Bacillus anthracis*. Mangiferin is a natural C-glucosylxantone compound that has many substantial curative potentials against numerous illnesses including cancers. Similarly, the anti-cancer effects of curcumin and its analogs have caught many enthusiasts over the last two decades. Screening antibacterial properties of these two compounds, employing high-throughput screening, authors identified potential binding sites on *B. anthracis*. Molecular docking revealed that curcumin and mangiferin, when synergistically combined, exhibited strong binding affinities at different sites on the bacterium.

The intricate relationship between cancer and bacteria has garnered increasing attention in recent years. For example, the gut microbiome is implicated in the pathogenesis of colorectal cancer (CRC), but the full scope of this dialogue is still unknown in 2024. While traditional cancer research has primarily focused on tumor cells and genetic mutations, emerging evidence highlights the significant role of microbial communities within the tumor microenvironment in cancer development and progression. The review Lu and Tong aims to provide a comprehensive overview of the current understanding of the complex interplay between cancer and bacteria. By conducting a thorough analysis of the existing literature, Lu and Tong underscore the multifaceted and intricate relationship between bacteria and cancer. Understanding this complex interplay could pave the way for novel therapeutic approaches and preventive strategies in cancer treatment.

The gut microbiota, intensely intertwined with mammalian physiology, significantly impacts health, productivity, and reproductive functions. The normal microbiota interacts with the host through the following key mechanisms: acting as a protective barrier against pathogens, maintain mucosal barrier integrity, assisting in nutrient metabolism, and modulation of the immune response (Khan et al.). This review emphasizes the critical ecological roles of mammalian microbiota, highlighting their essential contributions to health, productivity, and reproductive success. By integrating human and veterinary perspectives, it demonstrates how microbial communities enhance immune function, metabolic processes, and hormonal regulation across species, offering insights that benefit both clinical and agricultural advancements

The following experimental article is also about microbiota structure, with a special focus on reproductive tract of cattle. The top three bacterial phyla in bovine reproductive tract were *Proteobacteria, Firmicutes*, and *Bacteroidetes*, accounting for more than 85%. From the vagina to the uterus, the relative abundance of *Proteobacteria* gradually decreased, while that of *Firmicutes* gradually increased (Teng et al.). These findings lay a foundation for a comprehensive understanding of the structure of the genital tract microbiota of cows and its regulatory mechanisms.

The interplay between gut microbiota and host health is crucial for maintaining the overall health of the body and brain. Lot of microorganisms are involved, such as *Akkermansia muciniphila* which seems a promising next-generation probiotic with clinical application prospects. Emerging studies have reported various beneficial effects of *A. muciniphila* including anti-cancer, delaying aging, reducing inflammation, improving immune function, regulating nervous system function, whereas knowledge on its roles and mechanism in infectious disease is currently unclear. In summary, Li et al. believe that *A. muciniphila* is a promising therapeutic probiotic that may be applied for the treatment of a variety of infectious diseases.

Beneficial microbes may also have socioeconomic applications through industrial biotechnology, with the production of various metabolites, or enzymes. Chitin and chitooligosaccharides have been widely applied in food-related fields, with biodegradable, biocompatible, nontoxic, antimicrobial, and antioxidant activities. Processing and biorefinery should still being improved and the article from Xie et al. refine the taxonomic description of *Rhodococcus indonesiensis* and investigates its application in converting chitin into chitosan. The chitin deacetylase (*Ri*CDA) activity of the strain T22.7.1^T^ was optimized, and the enzyme was isolated and purified from the fermentation products. Product analysis revealed that *Ri*CDA treatment increased the deacetylation degree (DD) of natural chitin to 83%, surpassing that of commercial chitosan.

The Research Topic has been an advocate in providing key insights and bridging the knowledge gaps in understanding the dynamics of plant-microbe interactions. It encouraged the submission of high-quality research articles and reviews covering the most recent advances in microbiology. We are pleased to note that our Research Topic has attracted contributions from many highly regarded researchers deeply involved for many years in Microbiology around the world, including from Bangladesh, China, Czechia, Egypt, France, Germany, India, Malaysia, Romania, Saudi Arabia, and the USA. We received 16 submissions, 10 of which were accepted (seven original research articles, three reviews) for publication after rigorous peer-review, with a total of 93 authors.

